# Adaptation strategies in transnational education: a case study of an australian Master of Health Administration Course offered to chinese managers

**DOI:** 10.1186/s12909-021-03097-6

**Published:** 2022-01-22

**Authors:** Chaojie Liu, Qunhong Wu, Zhanming Liang, Leila Karimi, J. Adamm Ferrier, Jane Sheats, Hanan Khalil

**Affiliations:** 1 School of Psychology and Public Health, La Trobe University, 3086 Melbourne, VIC Australia; 2 School of Health Management, Harbin Medical University, 150081 Harbin Heilongjiang, China; 3 College of Public Health, Medical and Veterinary Sciences, James Cook University, 4811 Townsville, Qld Australia

**Keywords:** Health Management, Management training, Training evaluation, China

## Abstract

**Background:**

Management decisions in health influence patient care outcomes; however, health management development courses in China are rare. This study aims to document and evaluate a transnational Master of Health Administration (MHA) course launched in 2000 for the benefit of Chinese health managers.

**Methods:**

A case study of the MHA program jointly run by an Australian university and a Chinese Medical University was conducted. We reviewed the development of the MHA course through a document analysis (key events recorded in achieves, minutes, and audits) followed by reflection (by two course coordinators), extracting key themes related to adaptative strategies. We then conducted a questionnaire survey of 139 graduates seeking their views on relevance, satisfaction and challenges associated with each subject within the course, the relevance of key management skills (as determined by the Australasian College of Health Service Management competency framework), and the impact of the course on their personal career trajectories. Chi-square tests identified differences in the responses by age, gender, pre-training position, and current workplace.

**Results:**

The curriculum pedagogy followed the principles of practice-based reflective learning. Research findings and student feedback shaped the curriculum design and subject content, to enhance management practices of the students. Survey participants expressed high levels of satisfaction and confirmed the relevance of all study subjects. Two subjects, health economics and data management, were perceived as being the most challenging. Of the ten management skills we assessed, relatively low self-rated confidence was found in “strategic thinking” and “planning”. Younger and less experienced graduates were more likely to report learning challenges (*p* < 0.05). Frontline managers were least likely to obtain promotion by changing employers (χ^2^ = 6.02, *p* < 0.05) or being seconded to another position (χ^2^ = 9.41, *p* < 0.01).

**Conclusions:**

This case study illustrates the suitability of cross-country partnerships in health management training, which offers opportunities for managers to systematically explore and acquire a comprehensive set of management skills applicable to their career needs. Opportunities for developing training aligned to career development opportunities are critical for attracting and developing a competent and well-prepared health service management workforce in China.

**Supplementary Information:**

The online version contains supplementary material available at 10.1186/s12909-021-03097-6.

## Background

Management decisions in health influence patient care outcomes. Empirical studies show that both structural and procedural interventions by health managers have profound impact on the safety and quality of patient care [1–4]. In a study of 300 hospitals in nine European countries, Aiken et al. [5] found that that an increase of one patient to a nurse’s workload was associated with 7% increase in mortality for surgical patients 30-day post-admission, whereas for every 10% increase in the quantity of bachelor prepared registered nurses there was a corresponding decrease of mortality. A systematic review found that, in the United States, the discharge of patients from hospitals on weekends is associated with increased risk of readmission to the hospital [6]. There is increasing empirical evidence in the literature to support the link between human resources management measures and patient care outcomes [7]. An example is the “high performance work system” (HPWS), which advocates for a participatory approach to management that empowers employees and has demonstrated positive effects on patient care outcomes [8]. Despite this, in some circles the HPWS is criticised for shifting too much onus onto employees, leading to increased work pressure and potential negative health impacts on health workers [9].

Managers in health organisations face different challenges due to the context of the system in which they operate [10, 11]. Health administration and management is a relatively new discipline that aims to prepare health managers to address complex and ever-changing needs they encounter in their respective health system [11, 12]. In a planned economy, such as China prior to the late 1970s, health facility managers received directives from government authorities and implemented whatever they were told to do. The opening up and rapid development of the health care market in China since the 1980s involved the introduction of the private sector. This coincided with a dramatic reduction of governmental financial support to public facilities. Health service managers, predominantly from a clinical background and perhaps for the first time in their careers, were confronted with intense market competition and many found themselves ill prepared to address the realities of managing health services in a limited market economy [13]. Management competencies for health attracted attention, and in the 1980s, the Chinese Ministry of Health accordingly established 13 training centres for health services management. A health economic research network was established with support from the World Bank [14]. The Ministry of Health emphasises the importance of health management training and actively encourages clinician managers working in the health industry to undertake formal management training [15].

Despite this, most health managers in China who have a clinical background do not intend to pursue a life-long career in management [13, 16]. This is particularly the case for senior managers in the public sector, who are appointed by the governments with an official rank and have limited ability to move to a position in other organisations. Public sector senior health management positions have a strictly limited tenure in China. Managers originating from clinical roles must maintain clinical proficiency [16].

A Master of Health Administration (MHA) program situated within a medical university (usually in the school of public health) is appealing to the non-career health managers in China: unlike a Master of Business Administration (MBA) program, a MHA offers a master’s qualification in a “health-related discipline”. This qualification is well recognised by various health settings in China.

However, the development of postgraduate professional training programs in health administration has not featured similar growth compared to other disciplines [17]. In China, MBA courses are perhaps the most widely available degree program for managers as a consequence to the rapid industrialisation from the 1980s onwards [18], but for historical reasons, MBA courses in China rarely align with the health industry [19]. Despite a recent move to integrate medical universities within comprehensive universities [20], many medical universities remain as separate entities. These have often established Master of Public Administration (MPA) and Master of Public Health (MPH) programs [21]. In 2019, about 811,000 students commenced a course at master’s degree level in China [22]. A very small percentage of these postgraduate students (4,300 enrolments a year) were enrolled in public health related courses, and of these a little over half were 2,500 master’s students by coursework in public health and health administration per year. There is a conspicuous gap in supply: these graduates cannot possibly meet the needs of over one million (1,007,545) health institutions including 34,354 hospitals [23].

There is consensus in China regarding both the lack of and desirability of health management courses [12, 17]. However, there is paucity in the literature assessing relevance of the existing training courses for health management practitioners [15]. This may be attributed to an absence (to date) of a national health management competency framework [24], exacerbated by a failure to encourage experienced managers to transition toward an academic career [15, 25]. Some researchers criticised the professional training programs such as MPH and MPA for failing to differentiate themselves from research degrees [21].

In this paper, we conducted a case study on an established Australian coursework MHA program that was introduced to China in 2000. It is one of the earliest postgraduate professional degree training programs modified and specifically adapted for health managers in China. The course is aligned with the evolving competency frameworks developed by the Australasian College of Health Service Management (ACHSM). While the course is accredited by the ACHSM, studies into the validity of the ACHSM competency framework in the Chinese context are limited [24]. This study fills this gap in the literature by documenting the adaptation strategies adopted in the course from its inception to 2019 (prior to the outbreak of COVID-19), and seeking the views of recent graduates in relation to their perceived value of the course in relation to these management competencies. In a recent systematic review [26], Ebel and colleagues concluded that there is paucity in research into student outcomes despite it being a core procedure in competency- or outcome-based curricula.

Measures adopted in transnational education programs usually include licensing (selling curricula to another educational provider), articulation (recognition of teaching of other educational providers), and fly-in and fly-out delivery (teaching in an offshore location) [27]. To the best of our knowledge, the current study is one of the few, if any, documenting details of adaptation strategies in transnational education. It will help advance our understanding of good practices in cross-cultural and cross-system operations in health management training. Unlike MBA programs in which international principles and rules are preferred under the increasingly globalised environment, MHA courses tend to be highly contextualised simply because each country has its unique health system based upon historical development, cultural expectations and legal structures [12]. It follows then that contextual adaptation of health management training courses is a critical success factor.

This case study also offers a new perspective of transnational education based on the philosophy of student-centred education. This is extremely important in an increasingly globalised world of higher education. An evaluation of student experience from a student perspective not only informs educational providers improving curriculum design, but also contributes to advancing our understanding of health management training needs in general.

## Methods

### Study setting

China has more than 2,900 tertiary educational institutions; however, only 155 (5%) offer a degree program related to Public Health where health management training programs are traditionally located. Of the 828 institutions providing postgraduate degree training, 114 established a discipline in Public Health [19].

The MHA course evaluated in this study is a joint program run by an Australian university and a medical university in China. The course equates to “Health/Health Care Administration/Management” coded as 51.0701 in the classification of instructional programs (https://nces.ed.gov/ipeds/cipcode/cipdetail.aspx?y=56&cipid=91045) in the US. Student intake enrolment into this MHA course is limited by the Ministry of Education in China to 70 students per annum in recent years. All enrolled students were employed in the health industry upon commencement of study.

**Fig. 1:**
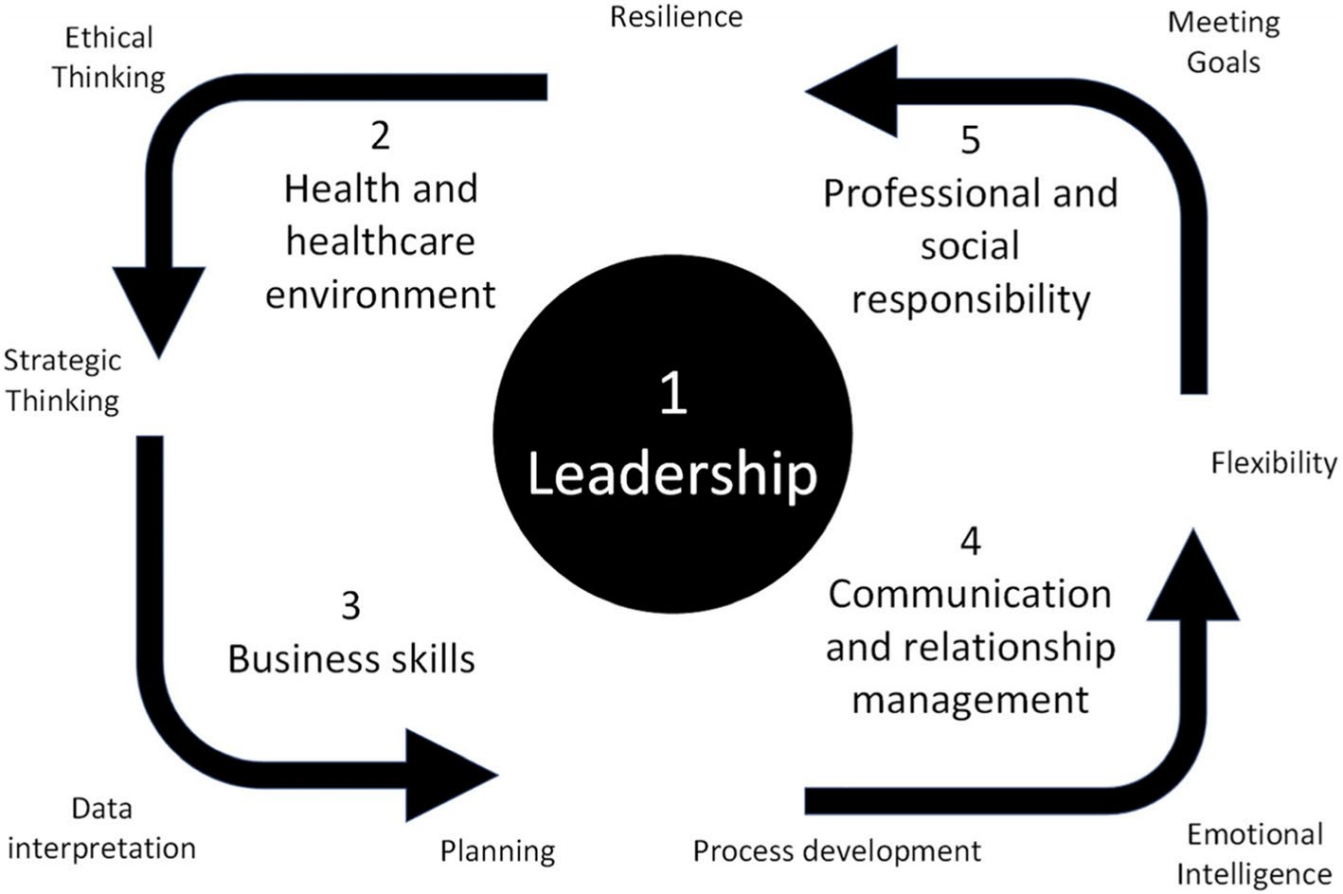
Competency Framework from the Australasian College of Health Service Management (adapted from https://achsm.org.au/education/competency-framework)

This joint course was adapted from the Australian practice-oriented MHA course guided by the ACHSM competency framework (Fig. 1). At the heart of the ACHSM model is the development and achievement in leadership, based on a strong foundational understanding of how health is achieved and the environment in which the health system operates. This alone is insufficient for effective management: health systems require managers who exhibit adept business skills, effective communication and relationship building capacities, and demonstrate their sensitivity to professional and social obligations in their daily lives.

While the majority of the Australian academics possess extensive policy and management experience, when the Australian practice-oriented curriculum was delivered in China, practice and contextual differences became apparent. The key issues that emerged included: (1) Chinese health managers were often subject to differing problems and priorities according to national structures [13]; (2) Few of the teaching academics in China had practical experience in management [15]; and (3) Engaging mature students in health management training courses is challenging. Most international courses encounter often unexpected difficulties in engaging student due to social, cultural and systemic differences [28, 29]. Adding to the complexity is the highly respect demanding status enjoyed by managers in terms of the traditional Chinese hospital culture [2]; (4) Management terms used in the two countries were different, with some English terms having no direct Chinese counterpart.

### Curriculum review

A comprehensive review of the curriculum, manner of instruction, and pedagogy of the transnational MHA program was conducted through document analyses and self-reflection. The first two authors (CL and QW) of this paper have been key academic members of the curriculum development since the very beginning of the program. They identified a list of key events over the period from the inception of the program to 2019: bilateral curriculum design workshops in 1999 and 2000; teaching and research agreements (renewed every three or five years); faculty appointments and development; academic exchange and joint research projects; anniversary celebrations (2010 in Beijing and 2011 in Harbin); government audits from Australia and China; program and alumni awards; and student and staff feedback. Minutes and reports related to these events were retrieved. CL reviewed these documents along with the curriculum operations manuals and subject guidelines and extracted key themes relevant to the four adaptation goals, including the underlying pedagogical philosophy guiding the curriculum development and the steps involved in adaptation of the course. The extracted themes were confirmed by QW and endorsed by all of the authors.

### Evaluation design and study participants

We conducted a cross-sectional survey of recent graduates from the MHA course over the three month period commencing in January and closing in April 2020. Study procedures were reviewed by the two partner universities and approved by the La Trobe University Human Research Ethics Committee (HEC20017). All methods were carried out in accordance with relevant guidelines and regulations. Informed consent was obtained from all subjects involved in the study: participants indicated consent at the start of the online questionnaire after reviewing a written description of the risks and benefits of study participation.

Invitations were sent via the WeChat groups of those who completed the degree course over the past three years (2018–2020). The invited WeChat groups comprised 206 eligible members from recent graduates, 128 (62%) of whom submitted a valid questionnaire. Eleven additional questionnaires were completed by those who graduated prior to 2018, resulting in a final sample size of 139.

#### Questionnaire

The evaluation questions focused on the practical values to the students, the highest level of Bloom’s taxonomy [30]. The evaluation indicators covered “reaction, learning, behaviour and results” in line with Kirkpatrick’s training evaluation framework [31]. A 25-item questionnaire (Supplementary File 1) was developed using Qualtrics by the research team. Respondents were asked to rate importance of key management skills, and the degree of relevance, satisfaction and challenges (reaction) of each study subject on a five-point Likert scale. The survey also captured data in relation to the demographic characteristics and management background of the respondents, the degree to which their obtained skills (learning) from the MHA course helped them to meet expectations in various managerial tasks (behaviour), and their career progression (results) after completion of the study. Respondents were also encouraged to provide recommendations through an open-ended question for further improvement of the MHA course.

The management skills assessed in this study were based upon the ACHSM health management competency framework (Fig. 1). We considered the attributes associated with this framework and so structured enquiries into the graduates’ self-assessment of their capacities in the following areas: emotional intelligence; leadership; resilience; data interpretation; flexibility; strategic thinking; ethical thinking; meeting goals; planning; and process development.

### Data analysis

Documents and qualitative data obtained from the open-ended question were coded by the first author CL. Common themes in relation to the four adaptation goals were extracted through categorising the codes and exploring links among the categories. The coding and extracted themes were reviewed by QW. Discrepancies, if any, were resolved by consulting with the lecturers involved in this program.

Quantitative data were exported from Qualtrics to a SPSS (IBM SPSS Statistics 26) dataset and analysed using descriptive statistics. Non-parametric tests such as Chi-square tests were performed to identify differences in the responses by age, gender, pre-training position, and current workplace. A *p* value of < 0.05 was considered statistically significant. A casewise approach was adopted to manage the small number of items containing missing values.

## Results

### Curriculum, Instruction, and Pedagogy

**Fig. 2:**
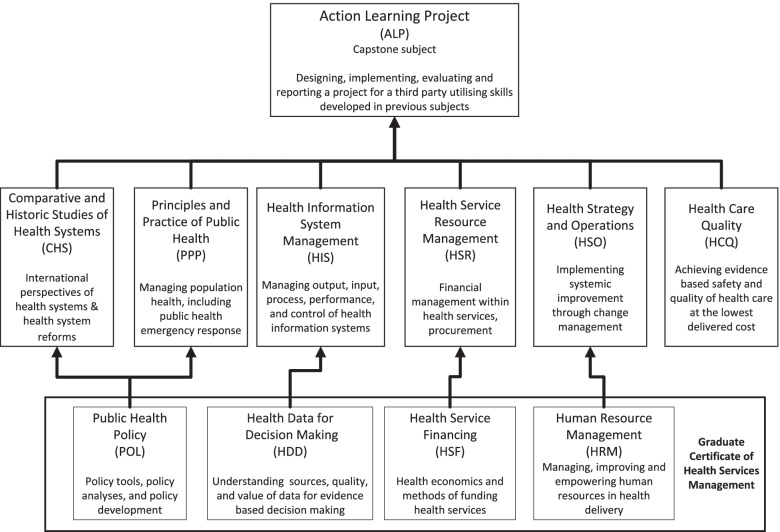
Subject composition of the Master of Health Administration (MHA) course

The course structure evaluated in this study resembles the Australian MHA course founded upon the same set of intended learning outcomes (Supplementary File 2), although the selection of teaching subjects and contents of these subjects consider the context of the Chinese health system. Overall, the course structure has remained stable in order to meet the ACHSM accreditation criteria based on its management competency framework. The current course contains 11 subjects (180 credit points) taught over a two-year study period. Students first need to develop a thorough understanding of management essentials, including workforce (HRM: Human Resource Management), policy (POL: Public Health Policy), information (HDD: Health Date for Decision-making), and health economics and financing (HSF: Health Sector Financing) before learning how to strengthen their management competencies through further study. For those who carry on with the masters program, each of the foundation subjects leads to others that build on these topics in relation to health systems (CHS: Comparative and Historic Study of Health Systems), population health (PPP: Principles and Practice of Public Health), information management (HIS: Health Information System), facility resource management (HSR: Health Service Resource Management), organisational management (HSO: Health Strategy and Operations), and safety and quality improvement (HCQ: Health Care Quality). The capstone subject Action Learning Project (ALP) adopts a reflective learning strategy [32], requiring students to improve their customary management skills, behaviours and practices through a supervised project utilising self-reflection (Fig. 2).

**Fig. 3:**
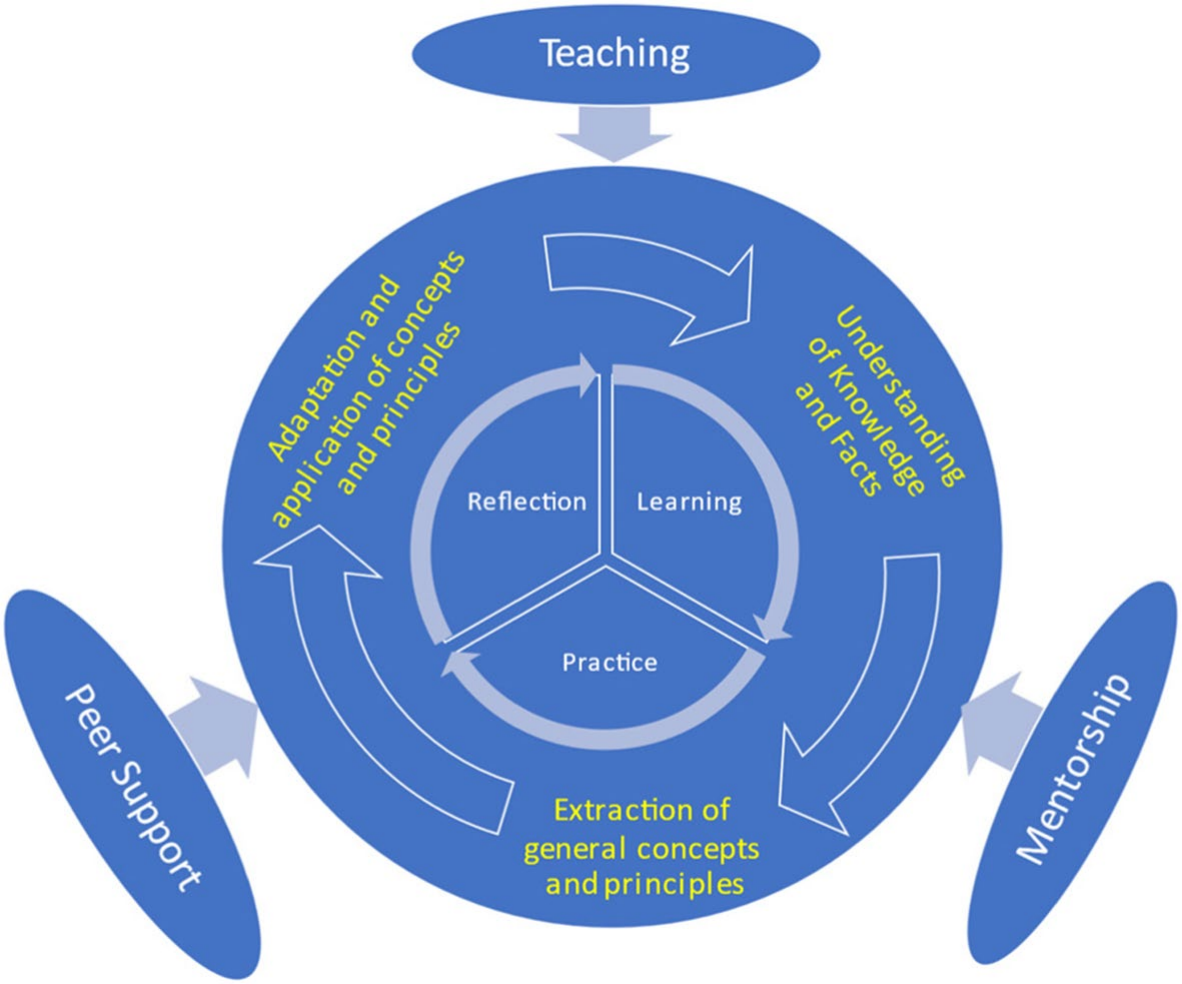
Pedagogical Framework

Teaching strategies and learning content were adapted to meet the needs of those working in the Chinese health system. The adaptation strategy is innovative in its research-informed approach [13, 15, 16] characterised by strong partnership, joint academic efforts with complementary expertise and skills, student-centredness and bilingual delivery, and reflective learning through management practices and participation in health reforms (Fig. 3). The design and delivery of the program also follows the principle of continuous improvement. Four steps were involved in development of the curriculum: (1) maintain essential course requirements in line with the management competency framework in Australia driven by the intended learning outcomes; (2) development of subject guidelines that adapt to the contextual needs of the Chinese health system through research and joint teaching workshops; (3) development and continuous revision of subject contents from an international perspective tailored to the needs of Chinese students; (4) update course contents throughout teaching cycles informed by stakeholder feedback. Indeed, the evaluation study reported in this manuscript is considered part of the continuous improvement process. Further details about the steps taken in adaptation of the course can be found in the supplementary file (2).

Teaching was delivered jointly by academics from both Australia and China. These co-teaching arrangements not only minimised potential language barriers, but also ensured adaptation of management concepts and tools to the Chinese context. Benefits flowed both ways: the experience for Australian teachers was enriching and benefitted their domestic teaching. All subjects were taught through three intensive blocks over a one-year period. Students were assigned into groups, each comprising up to 10 members including both experienced and less experienced managers. Group members were encouraged to support each other. The group leaders maintained close contacts with their academic supervisors and organised regular academic and social activities during and between study blocks.

## Findings of survey

### Demographic characteristics of study participants

About 64% of the respondents were female and 66% were younger than 41 years. The majority (63%) worked in a public facility, compared with 17% in government departments, 17% in universities, and 4% in the private sector (Table 1).

**Table 1:** Demographics of study participants (n = 139)

Characteristics		N	Percentage (%) of respondents
**Gender**	Female	89	64.0
	Male	50	36.0
**Age (years)**	< 36	44	31.7
	36–40	48	34.5
	41–45	26	18.7
	> 45	21	15.1
**Background prior to commencing MHA (can be multiple answers)**	Frontline managers	38	27.3
	Middle/department managers	31	22.3
	Senior managers	6	4.3
	Medicine/surgery	22	15.8
	Nursing/midwifery	21	15.1
	Other clinical roles	26	18.7
	Public health/Regulatory agency	19	13.7
	Non-clinical roles	17	12.2
**Current work sector**	Public sector	87	62.6
	Universities	23	16.5
	Governmental departments	23	16.5
	Private hospitals	4	2.9
	Other	2	1.3

All of the study participants had a undergraduate degree in a health-related discipline prior to enrolling in the MHA course. At the commencement of their studies, 50% respondents occupied a frontline or middle managerial position while 4% served as a senior manager. More than 63% reported direct involvement in healthcare services: 15.8% as physicians, 15.1% as nurses, 18.7% as other clinicians, 13.7% as public health practitioners. Only 12.2% reported no involvement in direct delivery of health services.

### Perception of the subjects contained in the course

Table 2 describes the student experience of specific subjects with the course, where students were asked to rank the subjects according to degree of challenge (degree of difficulty), satisfaction, relevance at the time of taking the course, and relevance in their post graduate experience. “Health Sector Financing (HSF)” and “Health Data for Decision-Making (HDD)” were considered the most challenging subjects as rated at 63% and 61% respectively by respondents in terms of the degree of difficulty, yet HSF also was associated with high levels of satisfaction (72.6%). Conversely, “Health Care Quality (HCQ)” and “Comparative and Historic Studies of Health Systems (CHS)” were rated equally (74%) as the most satisfying and applicable subjects to the course. In addition, “Human Resources Management (HRM)” was rated in the top two of the most relevant (90%) and applicable (81%) subjects. “Public Health Policy (POL)” and “Comparative and Historic Studies of Health Systems (CHS)” were also rated in the top two of the most satisfying and relevant subjects.

**Table 2:** Quality and relevance of subjects undertaken

Rating category	Percentage of rating from respondents^a^	Ranking Order
**Top 5 challenging subjects (Extremely/very challenging)**	63.0% 61.5% 57.4% 56.3% 55.6%	1. Health Service Financing 2. Health Data for Decision Making 3. Public Health Policy 4. Health Strategy and Operations 5. Human Resource Management
**Top 5 subjects with high satisfaction (very satisfied)**	74.3% 74.1% 73.5% 72.8% 72.6%	1. Health Care Quality 2. Comparative and Historic Studies of Health Systems 3. Health Strategy and Operations 4. Public Health Policy 5. Health Service Financing
**Top 5 subjects with high (extremely and very much) relevance when studying**	91.9% 90.4% 88.2% 87.5% 87.4%	1. Public Health Policy 2. Human Resources Management 3. Health Information System Management 4. Principles and Practice of Public Health 5. Health Care Quality
**Top 5 subjects with high (extremely and very much) relevance in current career**	81.5% 81.1% 80.7% 78.4% 75.6%	1. Human Resources Management 2. Health Care Quality 3. Health Information System Management 4. Public Health Policy 5. Action Learning Project

^a^ top five subjects on which the study participants reported the highest percentages of “extremely/very challenging”, “very satisfied” and “extremely/very relevant”

Perceived challenges and relevance of subjects were associated with age. There were no statistically significant differences in these ratings across various gender and position groups. Compared with their older counterparts, the younger respondents (≤ 35 years) perceived a higher level (*p* < 0.05) of challenges in studying six (PPP, CHS, HSR, HIS, HCQ, ALP) out of the eleven subjects and a higher level (*p* < 0.05) of relevance of some subjects in studying (CHS, ALP) and in working (PPP, CHS, HSO, HIS, ALP). Higher levels of relevance of studying POL (χ^2^ = 9.97, *p* < 0.05), CHS (χ^2^ = 10.61, *p* < 0.05), and HCQ (χ^2^ = 10.96, *p* < 0.05) were rated by the respondents currently working in the public sector (health).

### Self-rating on key management skills

**Fig. 4:**
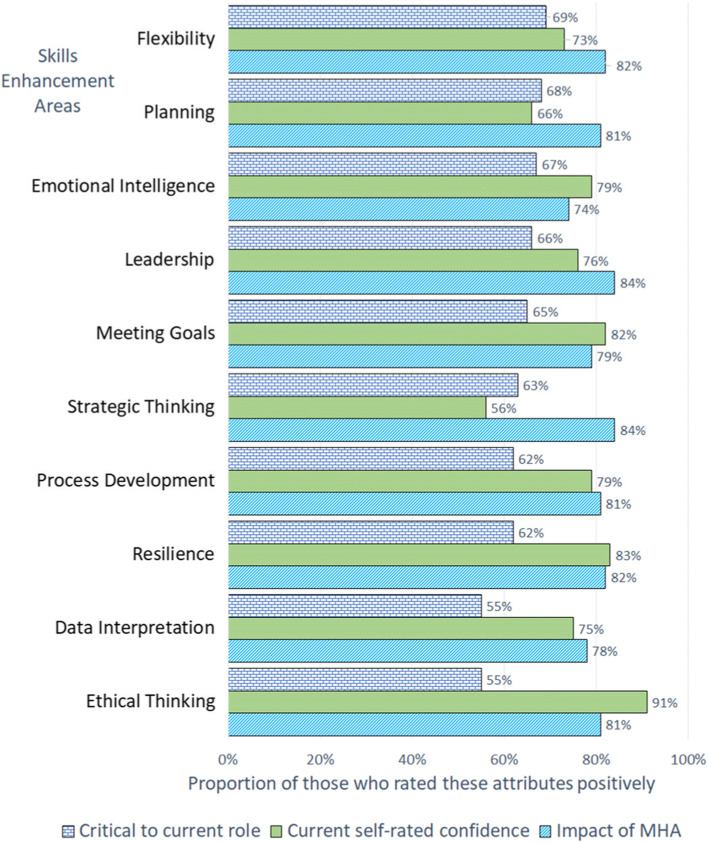
Management skills critical to current job, self-rated confidence, and enhanced by MHA (n = 139)

Figure 4 shows the overall skills that respondents indicated were enhanced by studying MHA. Leadership and strategic thinking were rated equally (84%), followed by flexibility (82%), resilience (82%), planning (81%), process development (81%), and ethical thinking (81%). It was noteworthy that emotional intelligence was received the lowest rating (74%). The ratings were consistent across the respondents with different characteristics.

The perceived importance of management skills was rated high consistently across the ten areas, with around 90% (ranging from 87 to 93%) considering them as essential for the performance associated with their current roles to “a great deal” or “a lot“. Flexibility was the skill with the highest percentage (69%) of respondents rating as being critical to “a great deal”, followed by planning (68%) and emotional intelligence (67%). Data interpretation and ethical thinking were rated at the bottom, both at 55% (Fig. 4). A higher level of importance of resilience (χ^2^ = 4.31, *p* < 0.05) was rated by the female respondents in comparison with their male counterparts. Those who did not occupy a managerial position gave lower ratings on importance of leadership (χ^2^ = 7.26, *p* < 0.05) and strategic thinking (χ^2^ = 6.60, *p* < 0.05).

Overall, high levels of confidence in management skills were found in the self-rating of the respondents (Fig. 4). Most described themselves as “extremely” or “very much” having the quality of ethical (91%), resilience (83%), and meeting goals (82%). A relatively lower rating on self-confidence was found in strategic thinking (56%) and planning (66%). The male respondents reported higher levels of self-confidence in data interpretation (χ^2^ = 6.37, *p* < 0.05) and strategic thinking (χ^2^ = 7.53, *p* < 0.05) compared with their female counterparts. Those working as a senior/middle manager (χ^2^ = 7.22, *p* < 0.05) found planning easier than others. No significant differences in self-ratings on confidence in management skills were found across various age and workplace groups.

### Satisfaction with MHA and areas of improvement

Participants were almost universally satisfied with the course (99%). No statistically significant differences in satisfaction ratings were found across various gender, age, and position groups.

**Fig. 5:**
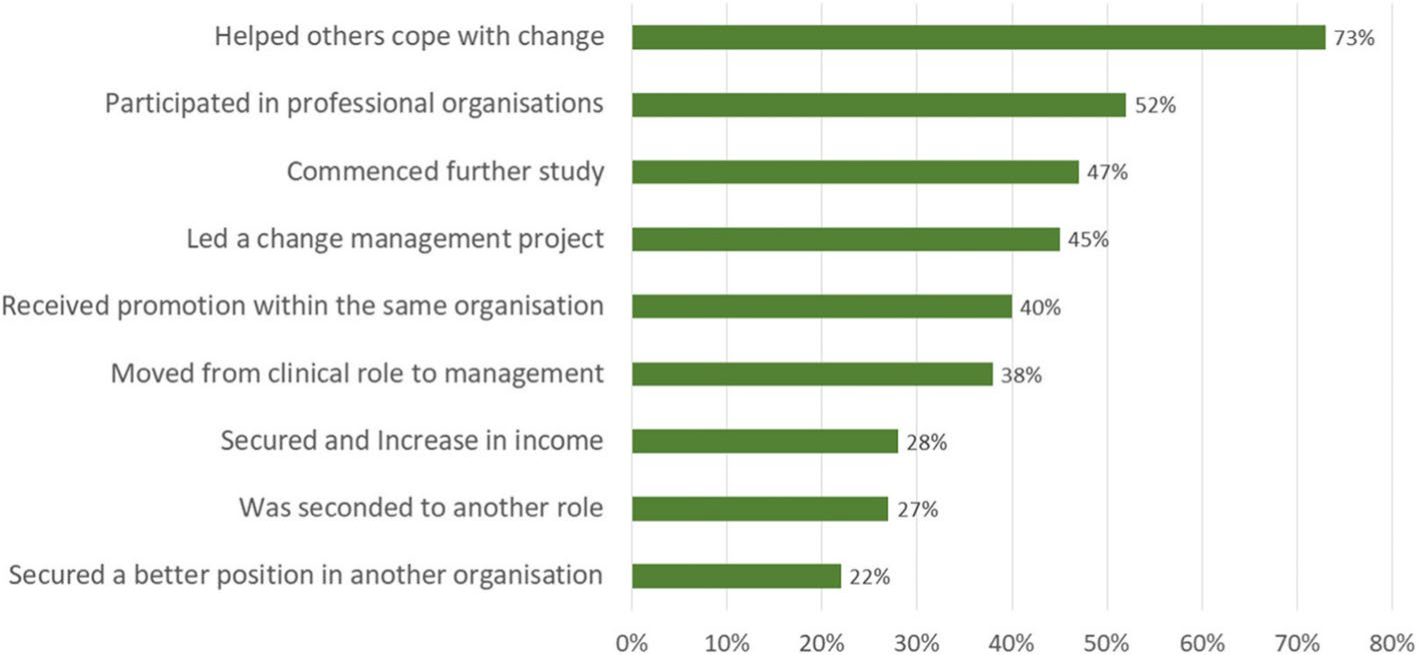
Milestone achieved after completion of MHA (n = 139)

The milestones achieved by the study participants are shown in Fig. 5. The highest milestone cited was “helping others to cope with change” (73%) whereas “securing a better position in another organisation” was the lowest selected milestone (22%), that said, “securing an increased income” was mentioned by 28% of all participants. Comparatively younger respondents (χ^2^ = 7.75, *p* < 0.05) were more likely to secure promotion through changing employers or via secondment to another position (χ^2^ = 8.03, *p* < 0.05). Those who did not occupy a managerial position prior to the study were more likely to get promoted (χ^2^ = 17.55, *p* < 0.001). In contrast, frontline managers reported the lowest likelihood to secure promotion through changing employers (χ^2^ = 6.02, *p* < 0.05) or being seconded to another position (χ^2^ = 9.41, *p* < 0.01).

More than 80% of respondents reported meeting management goals in relation to patient care and organisational development to or exceeding their expectations, except for the financial condition of the organisation (77%). Those of a comparatively higher age group were found to be associated with higher levels of perceived achievements in improving quality of patient care (χ^2^ = 15.54, *p* < 0.05). The senior/middle managers (χ^2^ = 15.10, *p* < 0.05) and those working in the public sector (χ^2^ = 14.53, *p* < 0.05) reported higher levels of achievements in patient safety. Working in the public sector (health) was also associated with higher ratings on process improvement (χ^2^ = 12.71, *p* < 0.05).

Table 3 shows the qualitative comments cited about the characteristics of MHA and explored student perceptions for improvement opportunities. Four key thematic categories emerged from the answers to the open-ended questions in relation to (1) needs-based course planning, (2) flexible delivery, (3) expanded career pathway and (4) extended impacts. The respondents recommended further strengthening of practice-based learning, strategic thinking and leadership, research skills, and public health emergency responses. Meanwhile, they demanded increasing flexibility in teaching and learning. One respondent argued that since both the backgrounds and career plans of students are highly diverse, this illustrates the importance of personalised training. Indeed, some respondents expressed an intention to build a pathway to higher degree courses and recommended the enhancement of research training, which was not a major focus of the MHA course.

**Table 3:** Major themes extracted from the open-ended question

Theme	Category	Suggested areas to be strengthened
Needs-based planning	Practice-based learning	Study tours to Australia; Field visits to health facilities; More hand-on practices; Improved integration of theories into practices.
	Strategic thinking and leadership	Strategy; Implementation with a clear goal and aim; Strategic decisions; Policy leadership, technical support, social mobilisation and participation.
	Research skills	Research components; Research methods for non-academics; Big data.
	Public health emergency response	Public health emergency response; Preparedness; Global public health emergency; Crisis management.
Flexible delivery	Delivery mode	More face to face time; Student-lecturer interactions; Diverse modes; More online delivery; More case studies; Individualised training plan; More guest lectures.
	Timetabling	Flexible; shortened blocks; Longer blocks.
Expanded career pathway	Doctoral program	Progress to doctoral course.
Extended impact	Quantity	More similar courses; Wider access.
	Quality	Flow-over effect; Excellent.
	Structure	Just right; Good structure.

## Discussion

Overall, the Australian MHA course has been successfully adapted to meet the needs of those working as Chinese health managers. Its research-informed development ensured high relevance of the teaching content. The bilingual delivery of the course not only eradicated language barriers of the students empowering them to actively engage in learning, but also ensured correct mapping and interpretation of the management lexicography. Chinese academics were supported by their Australian counterparts with complementary skill sets (in particular in management experience). The survey of recent graduates showed that the approach taken in this conjoint program was highly appreciated by the study participants, particularly in relation to its practice-based approach. They confirmed high level of relevance of the subject contents, including the management competency framework adapted from Australia to health managers in China; which is consistent with findings from the management competency studies conducted by Liang et al. [24, 25]. The study participants also expressed their high level of satisfaction in the learning experience and learning outcomes.

We are cautious to note that the student profiles in the conjoint MHA course are relatively homogenous as they all had a cognate qualification in health on entry to the course. This may explain why the study participants identified health economics and financing to be the most challenging subject offered in the course. This subject is perhaps the least familiar topic for them, having perhaps avoided previously exploring these issues throughout their careers as not being germane to their role and perhaps misconstrued as being perceived as critical of the role of the government. By contrast, the skills and issues explored in human resources management and health services quality management are closest to the everyday tasks of managers [16]. Indeed, the issues covered in both these subjects were rated as being the most relevant in their post graduate careers.

Internationally, it is a common challenge to meet the needs of each and every student as their needs are often highly diverse and contextually based [33]. We found in this study that the strong orientation towards practice in the MHA course imposed a greater level of challenges for the young and those with limited management experience. MHA graduates also reported relatively lower levels of self-confidence in strategic thinking and planning despite a high level of enhancement by the MHA course. This result is further supported by the qualitative data: strategic thinking and leadership emerged as one of the major areas for improvement of the course. This is unsurprising given that most students occupied a frontline position in a traditionally hierarchical management structure. Perceptions are usually contextual. In this study, for example, public health emergency response capacity also emerged as a major concern of the study participants from the qualitative data, possibly because this study was conducted during the COVID-19 pandemic in 2020.

Findings of this study provide useful evidence to guide further improvement of the course. For example, Emotional Intelligence (EI) was rated lowest in the ranking of the management skills enhanced by the course while it was rated amongst the top three critical managements skills required to the current graduates’ employment. The benefits of EI training for higher quality of care delivery is well demonstrated in the literature [34, 35]. The literature on EI support the notion that EI is a trainable skill that may well lead to higher performance [35, 36]. Given the training includes positive personal changes, improved connection with others, and the acquisition of important tools and skills it is critical that this area of MHA skill competency better implemented in the course.

Findings of this study also have some practice implications for transnational education. MHA belongs to adult education. The transformation learning theory is perhaps the most influential theory in adult education, which considers perspective transformation as the ultimate goal [37, 38]. The involvement of Australian lecturers and the international contents covered by this MHA course are not only additive to the teaching delivered by the Chinese academics, but also stimulative. It aims to expose students to different views and approaches and their underlying cultural roots with an expectation to trigger critical thinking from students on the validity of presuppositions that have shaped their perceptions and actions. According to the situated cognition theory [33], past experiences and work environment have a significant impact on adult learning. The MHA course evaluated in this study encourages students to learn in real-world scenarios as “they act in situations and are acted upon by situations” [37]. This reinforces a feeling of relevance from the MHA students about the Australian course, the management theories and management competency requirements embedded in the course.

Appropriate health management workforce policy is critical for maximising the value of MHA training courses. The relatively fewer promotion opportunities reported by the frontline managers in this study merits some policy attention. In China, most health workers seek life-long full-time employment within the one organisation [2] and promotion by changing employers is comparatively rare in China as reflected in this study. This is profoundly different to health employment in the west. Given that only a small proportion of health managers commit to formal management training [24, 25] even in the Australian system [39], practice-based learning becomes extremely important. Previous studies showed that senior executives of public hospitals in China tended to increase their participation in informal management-related training after taking up a management position [24, 25], only to find it difficult to commit sufficient time for study and learning. Evidence from different industries and healthcare systems in the past two decades confirms that management competence can be acquired and improved through targeted training programs and continuous professional development [40–42]. However, universities should tailor their education strategies towards meeting developmental needs within work environments through partnerships with health care organisations [43]. This would have the dual benefit of not only providing an opportunity to enhance employee skills capital but also denote an employer of choice. In China, this may also offer opportunities for some health managers to take up an academic role.

Further studies into the needs of and approach to differentiating research and professional courses in China are needed. While there is a consensus that MHA is a professionally oriented skills development course, rather than a research course [30, 44], some participants of this study foresaw the need for a pathway towards a research doctoral degree. Such a vision may have been shaped by two major factors. First, postgraduate degree programs are translated in Chinese language as “postgraduate research programs” (another example of a lexicon mapping challenge). Research training is inevitably seen as an integral part of these courses. Many attempts to uncouple practice-based postgraduate degree programs from research pathways have fallen short of expectations in China [21]. Second, a strong research component in MHA programs may help students in their career development not only in the managerial position but also after they resume clinical roles. In fact, professional promotions in China are traditionally biased toward research performance [16].

There are limitations in this study. Participants of this study were restricted to those who completed the MHA course in the past three years. Although this was a deliberate strategy to minimise recall bias, it reduced the chance of capturing post graduate career milestones of the participants. While our study found a high level of endorsement of the international course from the Chinese participants, we acknowledge a lack of comparison with other local courses due to difficulties in identifying a comparable practice-oriented postgraduate course in China. The evaluation study reported in this manuscript is a rapid appraisal contributing to continuous quality improvement. Data were limited to recent graduate self-reporting: we acknowledge more objective data over a longer period of observation are required to strictly follow the Kirkpatrick model (context, input, process, and product) [45]. Further studies are needed to evaluate the long-term impacts of MHA courses, in particular at the organisational and system levels, a conspicuous gap commonly existed in education evaluation studies [46].

## Conclusions

The current study documented the adaptation strategies and evaluated student learning outcomes of an Australian MHA course delivered in China. The findings offer insight into the relevance and applicability of MHA training programs to health management practices, particularly with respect to the context of the Chinese health system.

The Australian MHA program based on the ACHSM competency framework was adapted through a series of research and bilateral curriculum design workshops. It remains subject to continual improvement informed by student and staff feedback and external reviews and audits. The course was well received by health managers in China, which offered an opportunity for managers to systematically learn a comprehensive set of management skills that are applicable to their functions and roles. This case study also illustrates the suitability of cross-country partnerships in health management training. We recognise there is no one-size-fits-all solution to the design and delivery of MHA courses. Health managers usually have diverse backgrounds, which can be a valuable asset to the health care organisations. High levels of flexibility and specialisation are essential to MHA course design to meet diverse needs of health management students. This is particularly important in the MHA courses that aim at the international market: a common shortfall of the provision of “foreign” management courses is an overemphasis on theory lacking emphasis on practical, local-specific problem-solving strategies [10].

Health management training for young and inexperienced health workers is challenging as they naturally have less transferrable knowledge and expertise compared to their senior counterparts. The sharing of experience can serve as a driving force for reflection and learning [47]; but imposing enrolment restrictions will only discourage young people to pursue a management career, potentially exacerbating the shortage of health management workforce. Mentorship support based on the transformation learning theory is perhaps a good strategy to improve the learning experience of the young and inexperienced [48]. It is important to recognise the individuality of the adult learner [47]. Educators need to move away from didactic teaching, challenge their philosophical orientations in teaching, and embrace “behavioural, liberal, progressive, humanistic, and radical approaches to adult learning” [37].

## Supplementary Information

**Additional file 1.**

**Additional file 2.**

## Data Availability

The datasets used and/or analysed during the current study available from the corresponding author on reasonable request.

## Abbreviations

ACHSM: Australasian College of Health Service Management
ALP: Action Learning Project
CHS: Comparative and Historical Study of Health Systems
EI: Emotional Intelligence
HCQ: Health Care Quality
HDD: Health Date for Decision-making
HIS: Health Information System
HPWS: High Performance Work System
HRM: Human Resource Management
HSF: Health Service Financing
HSO: Health Strategy and Operations
HSR: Health Service Resource Management
MBA: Master of Business Administration
MHA: Master of Health Administration
MPA: Master of Public Administration
POL: Public Health Policy
PPP: Principles and Practice of Public Health
