# Correction to: Adaptation strategies in transnational education: a case study of an australian Master of Health Administration Course offered to chinese managers

**DOI:** 10.1186/s12909-022-03161-9

**Published:** 2022-02-23

**Authors:** Chaojie Liu, Qunhong Wu, Zhanming Liang, Leila Karimi, J. Adamm Ferrier, Jane Sheats, Hanan Khalil

**Affiliations:** 1 School of Psychology and Public Health, La Trobe University, Melbourne, VIC 3086 Australia; 2 School of Health Management, Harbin Medical University, Harbin, 150081 Heilongjiang China; 3 College of Public Health, Medical and Veterinary Sciences, James Cook University, Townsville, Qld 4811 Australia

**Correction to: BMC Med Educ 22, 52 (2022)**

**https://doi.org/10.1186/s12909-021-03097-6**

Following publication of the original article [1], we have been informed that author Zhanming Liang has been incorrectly affiliated.

The author affiliation has been updated above and the original article [1] has been corrected.
