# Supplementary material for: Adaptation strategies in transnational education: a case study of an australian Master of Health Administration Course offered to chinese managers
Source: BMC Med Educ. 2022 Jan 22;22:52. doi: 10.1186/s12909-021-03097-6 (PMC8783661; doi:10.1186/s12909-021-03097-6)
Supplement: Supplementary file 1 — Additional file 1. [file 12909_2021_3097_MOESM1_ESM.pdf]

Supplementary File 1: Questionnaire
Alumni Evaluation of the La Trobe University Master of Health Administration

Start of Block: Participant Information Statement

Q1.1

Alumni Evaluation of the La Trobe University Master of Health Administration

The following researchers will be conducting the study:

Role	Name	Organisation
Chief Investigator	A/Professor Hanan Khalil	La Trobe University, Department of Public Health
Associate Investigators	Professor Sandra Leggat	
	A/Professor Leila Karimi	
	Professor George Liu	
	Dr Zhanming Liang	
	Mr Adamm Ferrier	
External Investigator	Professor Qunhong Wu	Harbin Medical University
**Research funder**	This research is supported in kind by La Trobe University.	

What is the study about?

We warmly invite you to participate in an evaluation study of Master of Health Administration (MHA). We hope to learn about your experience of undertaking the course and its applicability in the workplace.

Do I have to participate?

Your contact details were obtained from the Alumni and Advancement Office of La Trobe University. Taking part of this study is voluntary. If you want to be part of the study we ask that you read the information below carefully. You can read the information below and decide at the end if you do not want to participate. If you decide not to participate this won't affect your relationship with La Trobe University.

Who is being asked to participate?

You have been asked to participate because you are an alumnus of La Trobe who completed the MHA.

What will I be asked to do?

If you want to take part in this study, we will ask you to complete an anonymous survey. It will take about 15 mins of your time to be part of this study.

What are the benefits?

There are no direct benefits to you in completing this questionnaire. However, we believe that your insights will help us to ensure that the course remains relevant and responsive to the real needs of the healthcare industry.

The benefits to the community is that we we hope the outcomes will identify strengths of the course and areas where we can improve.

What are the risks?

With any study there are (1) risks we know about, (2) risks we don't know about, and (3) risks we don't expect. If you experience something that you aren't sure about, please contact us immediately so we can discuss the best way to manage your concerns.

Language	Name/Organisation	Position	Telephone	Email
English	A/Prof Hanan Khalil, La Trobe University	Course Coordinator	03 94798802	h.khalil@latrobe.edu.au
Mandarin	Prof George Liu, La Trobe University	Director China Health Program	+613 9479 1715	c.liu@latrobe.edu.au

We do not foresee any risks associated with this study. As all data collected will be non-identifiable, it will not be possible to match the participants to the data collected.

Q1.2 What will happen to information about me?

We will **collect** information about you in ways that will not *intentionally* reveal who you are. **store** information about you in ways that will not *intentionally* reveal who you are. **publish** information about your responses so as to ensure that individuals cannot be identified in any type of publication from this study. **keep** your information for five after the project is completed. After this time we will destroy all of the data collected.
The storage, transfer and destruction of your data will be undertaken in accordance with the Research Data Management Policy https://policies.latrobe.edu.au/document/view.php?id=106/.

Any personal information you disclose will be handled in accordance with applicable privacy laws, any health information collected will be handled in accordance with the Health Records Act 2001 (Vic).

Will I hear about the results of the study?

We will let you know about the results of the study by publishing the journal article via the researchers' La Trobe university profiles. We will also provide a summary of the outcomes via the same email we used to contact you.

What if I change my mind?

If you no longer want to complete the questionnaire, simply close the web browser.

If you change your mind after clicking on the 'Submit' button, we cannot withdraw your responses because we cannot link who you are with your questionnaire responses. Your decision to withdraw at any point will **not** affect your relationship with La Trobe University.

Q1.3 Who can I contact for questions or want more information?

If you would like to speak to us, please use the contact details below:

Language	Name/Organisation	Position	Telephone	Email
English	A/Prof Hanan Khalil, La Trobe University	Course Coordinator	03 94798802	h.khalil@latrobe.edu.au
Mandarin	Prof George Liu, La Trobe University	Director China Health Program	+613 9479 1715	c.liu@latrobe.edu.au

What if I have a complaint?

If you have a complaint about any part of this study, please contact:

Ethics Reference Number	Position	Telephone	Email
HREC 20017	Senior Research Ethics Officer	+61 3 9479 1443	humanethics@latrobe.edu.au

If you would like to download a copy of this participant information statement please click **on the link below**.

Participant Information Statement Alumni Evaluation of the La Trobe University Master of Health Admi

Q1.4 I understand the Participant Information Statement, and any questions have been answered to my satisfaction. I agree to participate in the study. I know I can withdraw at any time prior to submitting the survey by simply closing the browser. I agree that the information I provide may be included in presentations, internal reports and published in journals on the condition that I cannot be identified.

- ○ I agree to participate in the study
- ○ I would prefer not to participate

Skip To: End of Survey If Q1.4 = 2

End of Block: Participant Information Statement

Start of Block: Demographic Data

Q2.1 Please tell us your gender.

- ○ Male
- ○ Female
- ○ Non-binary/other
- ○ Prefer not to disclose

Q2.2 Which age group do you currently belong?

- ○ up to and including 30 years
- ○ 31-35 years
- ○ 36-40 years
- ○ 41-45 years
- ○ 46-50 years
- ○ 51-55 years
- ○ 56-60 years
- ○ 61-65 years
- ○ 66 years and above
- ○ Prefer not to disclose

Q2.3 What was your background **prior to commencing** the MHA? (Click as many as apply)

- ☐ Executive Management (CEO, CFO, COO, Board etc)
- ☐ Divisional Management (CMO, DON, etc)
- ☐ Frontline/ Unit Management (e.g. NUM, Chief Pharmacist etc)
- ☐ Medicine/Surgery Clinician
- ☐ Nursing/Midwifery Clinician
- ☐ Paramedicine
- ☐ Physiotherapy Clinician
- ☐ Occupational Therapy Clinician
- ☐ Speech Pathology Clinician
- ☐ Rehabilitation Counselling
- ☐ Pharmacy
- ☐ Dentist
- ☐ Podiatrist
- ☐ Prosthetics & Orthotics
- ☐ Orthopics
- ☐ Other clinical role (please advise)
- ☐ Aged Care
- ☐ Finance
- ☐ Non Clinical Support staff (eg CSSD)
- ☐ Logistics
- ☐ Regulatory authority
- ☐ Public Health
- ☐ Consultancy services
- ☐ Armed Forces (e.g. Army, Navy, Air Force, Marines)
- ☐ Correctional health (prison services)
- ☐ Private Health Insurance industry
- ☐ Other non clinical role (please advise)
- ☐ Prefer not to disclose

Q2.4 For what type of organisation do you currently work?
(choose one that best describes your direct employment situation)

- Public sector (health)
- Private sector (health)
- Self employed
- On extended leave (e.g. parental leave, extended travel, sabbatical)
- Public sector (non-health)
- Private sector (non-health)
- Retired from workforce
- Prefer not to disclose
- Higher education
- Central government
- Provincial government
- Municipal, county (district), township government
- Not currently working, seeking a new role

Q2.5 What would best describe your **present** role (Choose one)

- ○ Executive Management (CEO, CFO, COO, Board etc)
- ○ Divisional Management (CMO, DON, etc)
- ○ Frontline/ Unit Management (e.g. NUM, Chief Pharmacist etc)
- ○ Medicine/Surgery Clinician
- ○ Nursing/Midwifery Clinician
- ○ Paramedicine
- ○ Physiotherapy Clinician
- ○ Occupational Therapy Clinician
- ○ Speech Pathology Clinician
- ○ Rehabilitation Counselling
- ○ Pharmacy
- ○ Dentist
- ○ Podiatrist
- ○ Prosthetics & Orthotics
- ○ Orthopics
- ○ Other clinical role (please advise)
- ○ Aged Care
- ○ Finance
- ○ Non Clinical Support staff (eg CSSD)
- ○ Logistics
- ○ Regulatory authority
- ○ Public Health
- ○ Consultancy services
- ○ Armed Forces (e.g. Army, Navy, Air Force, Marines)
- ○ Correctional health (prison services)
- ○ Private Health Insurance industry
- ○ Other non clinical role (please advise)
- ○ Prefer not to disclose

Q2.6 What is the best description of the course you took?

- ○ La Trobe PGDHSM only
- ○ Other La Trobe graduate diploma or degree (please advise)
- ○ Postgraduate diploma or degree from other universities (please advise)
- ○ La Trobe MHA

Q2.7 In which year did you **commence study** for your post graduate diploma

____

In which year did you **complete study** for your post graduate diploma

____

Q2.8 In which year did you **commence study** for your master degree

____

In which year did you **complete study** for your master degree

____

Q2.9 What was your enrolment mode?

- ▢ Full time
- ▢ Part time
- ▢ It varied: some semesters were full time, others were not
- ▢ Block mode (intensive learning over a short period)
- ▢ Prefer not to disclose

Q2.10 What was the **main** delivery mode?

- ○ I attended classes in person
- ○ I attended most classes in person, but some were only offered online
- ○ I was a distance education student
- ○ I studied some subjects online, and some subjects in class

Q2.11 What is your **preference** for the delivery of subjects?

- ○ I prefer classes in person
- ○ I don't mind a mix, depends on the subject
- ○ I prefer online delivery
- ○ I actively dislike online classes

End of Block: Demographic Data

Start of Block: Level one: Participants reaction to content

Q3.1 How **challenging** did you find each of the subjects at the time you were studying the MHA?

	Extremely challenging	Very challenging	Moderately challenging	Slightly challenging	Not challenging	Not applicable	Did not enrol
Health Policy	○	○	○	○	○	○	○
Health Economics and Financing	○	○	○	○	○	○	○
Health Data for Decision Making	○	○	○	○	○	○	○
Human Resources Management	○	○	○	○	○	○	○
Principles and Practice of Public Health	○	○	○	○	○	○	○
Comparative and Historic Studies of Health Systems	○	○	○	○	○	○	○
Health Strategy and Operational Management	○	○	○	○	○	○	○
Health Resources Management	○	○	○	○	○	○	○
Health Information System	○	○	○	○	○	○	○
Health Care Quality	○	○	○	○	○	○	○
Action Learning Project	○	○	○	○	○	○	○
Comparative Health Management	○	○	○	○	○	○	○
Program Planning and Evaluation	○	○	○	○	○	○	○

Q3.2 How **satisfied** were you with each of the subjects at the time you were studying the MHA?

	Extremely satisfied	Somewhat satisfied	Somewhat dissatisfied	Extremely dissatisfied	Did not enrol in this subject
Health Policy	○	○	○	○	○
Health Economics and Financing	○	○	○	○	○
Health Data for Decision Making	○	○	○	○	○
Human Resources Management	○	○	○	○	○
Principles and Practice of Public Health	○	○	○	○	○
Comparative and Historic Studies of Health Systems	○	○	○	○	○
Health Strategy and Operational Management	○	○	○	○	○
Health Resources Management	○	○	○	○	○
Health Information System	○	○	○	○	○
Health Care Quality	○	○	○	○	○
Action Learning Project	○	○	○	○	○
Comparative Health Management	○	○	○	○	○
Program Planning and Evaluation	○	○	○	○	○

Q3.3 How **relevant** did you find each of the subjects at the time you were studying the MHA?

	Extremely relevant	Very relevant	Moderately relevant	Slightly relevant	Not relevant at all	Not applicable
Health Policy	○	○	○	○	○	○
Health Economics and Financing	○	○	○	○	○	○
Health Data for Decision Making	○	○	○	○	○	○
Human Resources Management	○	○	○	○	○	○
Principles and Practice of Public Health	○	○	○	○	○	○
Comparative and Historic Studies of Health Systems	○	○	○	○	○	○
Health Strategy and Operational Management	○	○	○	○	○	○
Health Resources Management	○	○	○	○	○	○
Health Information System	○	○	○	○	○	○
Health Care Quality	○	○	○	○	○	○
Action Learning Project	○	○	○	○	○	○
Comparative Health Management	○	○	○	○	○	○
Program Planning and Evaluation	○	○	○	○	○	○

Q3.4 How **relevant** do you find each of the subjects **at this point in your career**?

	Extremely relevant	Very relevant	Moderately relevant	Slightly relevant	Not relevant at all	Not applicable
Health Policy	○	○	○	○	○	○
Health Economics and Financing	○	○	○	○	○	○
Health Data for Decision Making	○	○	○	○	○	○
Human Resources Management	○	○	○	○	○	○
Principles and Practice of Public Health	○	○	○	○	○	○
Comparative and Historic Studies of Health Systems	○	○	○	○	○	○
Health Strategy and Operational Management	○	○	○	○	○	○
Health Resources Management	○	○	○	○	○	○
Health Information System	○	○	○	○	○	○
Health Care Quality	○	○	○	○	○	○
Action Learning Project	○	○	○	○	○	○
Comparative Health Management	○	○	○	○	○	○
Program Planning and Evaluation	○	○	○	○	○	○

Q3.5 **Overall**, how satisfied were you with your experience of undertaking the MHA?

- Extremely satisfied
- Somewhat satisfied
- Neither satisfied nor dissatisfied
- Somewhat dissatisfied
- Extremely dissatisfied

Q3.6 To what extent would you recommend the MHA to a colleague?

- Extremely likely
- Somewhat likely
- Neither likely nor unlikely
- Somewhat unlikely
- Extremely unlikely

End of Block: Level one: Participants reaction to content

Start of Block: Level two: Changes associated with learning

Q4.1 How would you **describe** yourself in terms of the following qualities?

	Extremely	Very much	Moderately	Slightly	Not at all	Prefer not to answer
I have strong emotional intelligence	○	○	○	○	○	○
I enable others to see different perspectives	○	○	○	○	○	○
I am resilient	○	○	○	○	○	○
I can interpret data	○	○	○	○	○	○
I am flexible	○	○	○	○	○	○
I am strategic	○	○	○	○	○	○
I am ethical	○	○	○	○	○	○
I can achieve challenges	○	○	○	○	○	○
I can plan easily	○	○	○	○	○	○
I understand process	○	○	○	○	○	○

End of Block: Level two: Changes associated with learning

Start of Block: Level three: application of skills and new knowledge

Q5.1 To what extent were the following **enhanced by undertaking the MHA**?

	A great deal	A lot	A moderate amount	A little	Not at all	Prefer not to answer
Emotional intelligence	○	○	○	○	○	○
Leadership	○	○	○	○	○	○
Resilience	○	○	○	○	○	○
Data interpretation	○	○	○	○	○	○
Flexibility	○	○	○	○	○	○
Strategic thinking	○	○	○	○	○	○
Ethical thinking	○	○	○	○	○	○
Meeting goals	○	○	○	○	○	○
Planning	○	○	○	○	○	○
Process development	○	○	○	○	○	○

Q5.2 Are you currently employed in the health sector?

○ Yes

○ No

Skip To: End of Block If Q5.2 = 2

Q5.3 How **essential** are each of the following in your current role?

	A great deal	A lot	A moderate amount	A little	None at all	Prefer not to answer
Emotional intelligence	○	○	○	○	○	○
Leadership	○	○	○	○	○	○
Resilience	○	○	○	○	○	○
Data interpretation	○	○	○	○	○	○
Flexibility	○	○	○	○	○	○
Strategic thinking	○	○	○	○	○	○
Ethical thinking	○	○	○	○	○	○
Meeting goals	○	○	○	○	○	○
Planning	○	○	○	○	○	○
Process development	○	○	○	○	○	○

End of Block: Level three: application of skills and new knowledge

---

Start of Block: Level four: organisational benefit

Q6.1 Since completing the MHA, to which extent have you achieved the following outcomes?

	Far exceeded expectations	Exceeded expectations	Met expectations	Short of expectation	No opportunity	Not relevant to role	Prefer not to answer
Improved quality of patient care	○	○	○	○	○	○	○
Improved patient outcomes	○	○	○	○	○	○	○
Enhanced patient safety	○	○	○	○	○	○	○
Identified process improvement opportunities	○	○	○	○	○	○	○
Enhanced clinical governance arrangements	○	○	○	○	○	○	○
Improved departmental/ organisation productivity	○	○	○	○	○	○	○
Improved financial security for an organization	○	○	○	○	○	○	○
Identified improvement opportunities for an organization	○	○	○	○	○	○	○
Identified or averted a developing risk	○	○	○	○	○	○	○
Leading people through a change process	○	○	○	○	○	○	○
Other (Please advise)	○	○	○	○	○	○	○

End of Block: Level four: organisational benefit

Start of Block: Level five: Return on investment

Q7.1 After completion of the MHA, which of the following professional milestones have you experienced or achieved?

	Four or more times	two or three times	Once	Not yet	Prefer not to answer	Not applicable
Received promotion within the same organization	○	○	○	○	○	○
Secured a better position in another organization	○	○	○	○	○	○
Moved from clinical role to management	○	○	○	○	○	○
Was seconded to another role	○	○	○	○	○	○
Secured an increase in income	○	○	○	○	○	○
Led a change management project	○	○	○	○	○	○
Helped others cope with change	○	○	○	○	○	○
Commenced further study	○	○	○	○	○	○
Participated in professional organisations	○	○	○	○	○	○
Other (Please advise)	○	○	○	○	○	○

Q7.2 Have you published your results of Action Learning Subjects?

- ○ Yes
- ○ Not yet, but plan to do so
- ○ No, deemed commercial secret
- ○ No
- ○ Other (please advise)
- ○ Prefer not to disclose

End of Block: Level five: Return on investment

Start of Block: Other issues

Q8.1 Do you have any suggestions for how we might otherwise improve the MHA for future students?

____

____

____

____

____

End of Block: Other issues
