# Supplementary material for: Adaptation strategies in transnational education: a case study of an australian Master of Health Administration Course offered to chinese managers
Source: BMC Med Educ. 2022 Jan 22;22:52. doi: 10.1186/s12909-021-03097-6 (PMC8783661; doi:10.1186/s12909-021-03097-6)
Supplement: Supplementary file 2 — Additional file 2. [file 12909_2021_3097_MOESM2_ESM.docx]

**Supplementary File** 2:

**Developing a Master of Health Administration (MHA) course adapted to the context of China**

The following steps were involved in developing the MHA course tailored to the needs of health managers in China. Please note that these steps intertwine each other, and over time the principle of continuous quality improvement has been applied. Adaptations are guided by the pedagogical framework that emphasises the need for health system reform and encourages student creativity and learning transference to new situations, rather than specific tools and tasks. Teaching was delivered in very intensive blocks while students were mentored to apply what they learnt to practice and shared experiences and obtained feedback in subsequent face-to-face study blocks

**Step one: maintain essential course requirements in line with the management competency framework in Australia**

This step was driven by the need for a management competency framework in China.

- Adopting the Australian course intended learning outcomes (CILOs, Table S1) on the understanding that it was critical to establish equivalency between onshore and offshore operations from the perspectives of both Australian and Chinese educational authorities.

| **Table S1. Course Intended Learning Outcomes** |
| --- |
| 1. Apply research principles and methods to critically appraise the health management body of knowledge and to managerial and leadership practice.  2. Synthesise information on population needs, health care delivery systems, funding and financing arrangements and government policy to create, revise, implement and evaluate evidence-based strategies that enhance population health and maximise organisational potential.  3. Apply project management tools and techniques underpinned by contemporary theoretical models to cultivate change.  4. Evaluate and synthesise the impact of health system and health organisation resource allocation decisions and apply to future resource allocation decisions.  5. Identify and assemble relevant data to analyse operational and clinical performance of the health care system or organisation to enhance patient care and population health outcome.  6. Develop and apply ethical evidence-based leadership skills directed to individuals and teams across diverse organisational environment.  7. Effectively communicate ideas and information to different audiences and diverse setting.  8. Apply critical reflective practice that adapts responsively to the needs of different contexts and stakeholder groups. |

- Identifying core subjects from the existing Australian MHA course that addressed all CILOs through Sino-Australian pedagogy workshops – electives are avoided to minimise complexity and costs. Unlike the current Australian MHA course structure, the China MHA does not have specialisation. The course corresponds to “Health/Health Care Administration/Management” recorded in the US classification of instructional programs (CIP 51.0701).
- Maintaining accreditation from the Australasian College of Health Services Management in line with its management competency framework.

**Step two: development of subject guidelines that adapt to the contextual needs of the Chinese health system**

- Building institutional linkages through a project funded by the Australian Agency for International Development.
- Enhancing academic communications and mutual understanding through bilateral workshops in China and in Australia – working with prestigious universities in China such as Peking Medical University, West China University of Medical Sciences, and Harbin Medical University.
- Establishing evidence-based training needs assessment – six doctoral research projects
- Likun Pei (1998). *Hospital management in a time of change: the need for management training (and policy reform) in three teaching hospitals in Yunnan*. Melbourne: La Trobe University
- Chaojie Liu (2003). *Closing the gap between policy and reality: a study of community health services in Chengdu and Panzhihua*. Melbourne: La Trobe University
- Hui Yang (2004). *Priorities and strategies for health information system development in China: how provincial health information systems support regional health planning.* Melbourne: La Trobe University
- Zhiping Gong (2004). *Developing casemix classification for acute hospital inpatients in Chengdu, China*. Melbourne: La Trobe University
- Hongwen Zhao (2005).*Governing the healthcare market: regulatory challenges and options in the transitional China.*Melbourne: La Trobe University
- Yan Zhang (2006). *Improving prophylactic antibiotic prescribing for surgeons in a general hospital in Beijing: lessons from a trial of localised antibiotic guidelines, drug utilisation study and education.* Melbourne: La Trobe University

**Step three: development and continuous revision of subject contents from an international perspective tailored to the needs of Chinese students**

- Engaging academics with diverse backgrounds – country (Australia, China, and other countries), qualification (medicine, nursing, pharmacy, public health, allied health, management, economics etc), expertise (research, practice).
- Training the trainers – teaching workshops, supervision of doctoral students from China, peer support
- Involving academics who enjoy a high research profile in China – for example those with books being translated into Chinese language.
- Co-authoring book chapters (e.g. “Health Policy in and for China” and “Working with Information” published in English and Chinese) and peer-reviewed articles with Chinese colleagues.
- Pairing academics from Australia and China to develop teaching and learning materials (presentations, small exercises, case studies, recommended readings) in English and Chinese languages
- Initiated in English if led by Australian academics
- Initiated in Chinese if led by Chinese academics

**Step four: update course contents throughout teaching cycles**

- Informed by the literature
- Informed by ongoing joint research
- Informed by regular student feedback
- Informed by alumni surveys
- Informed by collegial feedback, especially those Chinese academics who co-teach into the course
- Informed by active engagement in health services projects in China
